# Adder bite: an uncommon cause of compartment syndrome in northern hemisphere

**DOI:** 10.1186/1757-7241-18-50

**Published:** 2010-09-20

**Authors:** Lars H Evers, Tanja Bartscher, Thomas Lange, Peter Mailänder

**Affiliations:** 1 Department of Plastic, Hand-, Reconstructive Surgery, Burn Center, University of Lübeck, Germany

## Abstract

Snakebite envenomation is an uncommon condition in the northern hemisphere, but requires high vigilance with regard to both the systemic effects of the venom and the locoregional impact on the soft tissues. Bites from the adder, Vipera Berus, may have serious clinical consequences due to systemic effects. A case of a 44-year-old man is reported. The patient was bitten in the right hand. He developed fasciotomy-requiring compartment syndrome of the upper limb. Recognition of this most seldom complication of an adder bite is vital to save the limb. We recommend that the classical signs and symptoms of compartment syndrome serve as indication for surgical decompression.

## Background

Snakebites in northern Europe are a rare source of severe medical conditions including systemic effects and compartment syndrome. Nevertheless the Adder, Vipera Berus, is endemious in northern Europe and the only snake, which habits even in the arctic region [1]. It is a relatively small, thickbodied snake typically reaching a length of 65 cm as adults. The venom is produced by modified salivary glands and is injected 2-3 mm subcutanously into the victim. The venom is containing a complex mixture of high molecular weight proteins, mainly proteases, peptid hyaluronidase, and phospholipases whose effects are predominantly cytotoxic and hemorrhagic [1,2]. The cytotoxic component attacks the vascular endothelial linings, typically resulting in early and extensive edema and hypovolemia. Bruising also occurs and is usually most pronounced in the regions of the main lymphatic trunks and regional lymph nodes. Hypotension is the most important sign of systemic envenoming, usually developing within two hours [2,3]. Victims may feel faint, and children in particular may become drowsy or semiconscious. Nausea is usual and vomiting is a common and prominent feature, which may last for several days. Diarrhea may also occur. Other systemic effects include abdominal colic, incontinence, sweating, vasoconstriction, tachycardia, and angio-edema of the face, lips, gums, tongue, throat and epiglottis, urticaria and bronchospasm [2,3]. Systemic hemorrhage and coagulopathy appear to be rare in man, possibly because of the combination of relatively low venom potency and small delivered dose in a victim of relatively large body mass. Although it has not yet been isolated, there is some evidence that a cardiotoxic component is present in the venom causing T wave inversion, myocardial damage, and second degree heart block [4]. Laboratory test results include neutrophil leucocytosis, thrombocytopenia, initial hemoconcentration and later anemia resulting from extravasation into the bitten limb, and rarely hemolysis, elevation of serum creatine phosphokinase, and metabolic acidosis [3].

The description of the clinical symptoms can be classified into five envenomation grades, grade 0-4, serving as indicator for the need for antivenom treatment as well as prognostic estimator. The severity of the reaction to snakebites depends on the degree of envenomation. Downey, Omer and Moneim describe a system whereby, grade 0 means there is no envenomation and indicates swelling and erythema around the fang marks of <2.5 cm, grade 1 indicates swelling and erythema of 2.5 to 15 cm but no systemic signs, grade 2 indicates swelling and erythema of 15 to 40 cm with mild systemic signs, grade 3 indicates swelling and erythema of >40 cm with systemic signs, and grade 4 indicates severe systemic signs including coma and shock [5].

The incidence of severe adder bites (grade 3-4) in Europe is described with a mean of 0.6/1 million inhabitants per year with a peak in summer month [6]. The main site, where the bite occurred was the hand with 52%, followed by the foot with 38% [2]. Bites on the hand were usually on the thumb or fingers and often resulted from the person picking up a snake, while bites on the foot were most often on the ankle, and were the result of stepping on a snake. Snakebites usually happen accidentally. Men were more likely to be bitten than women or children, and incidents of adder bite have been recorded in people of all ages ranging from 1-78 years [7].

Although most adder (V. berus) bites result in trivial symptoms, envenoming can produce both local and systemic effects, which can cause death from 6 to 60 hours after a bite, particularly in children and the elderly [8]. The critical period for a victim is usually the first 12 hours after being bitten but may last for several days.

Compartment syndrome after an adder bite is extremely rare, but has been reported in the palm and forearm following envenomation [9-11]. Other more common reasons for compartment syndrome of the upper extremity include forearm fractures, ischemic-reperfusion following injury, hemorrhage, vascular puncture, intravenous drug injection, casts, prolonged limb compression, crush injuries and burns. Without prompt surgical treatment, it may lead to nerve damage and muscle death. Edema of the soft tissue within the compartment further raises the intra-compartment pressure, which compromises venous and lymphatic drainage of the injured area. Pressure, if further increased in a reinforcing vicious cycle, can compromise arteriole perfusion, leading to further tissue ischemia [12].

The normal mean interstitial tissue pressure is near zero mm Hg in non-contracting muscle. If this pressure becomes elevated to 30 mm Hg or more, small vessels in the tissue become compressed, which leads to reduced nutrient blood flow i.e., ischemia and pain. Of particular importance is the difference between compartment pressure and diastolic blood pressure; where diastolic blood pressure exceeds compartment pressure by less than 30 mm Hg it is considered an emergency [13]. The compartment pressure measurement can be helpful in the assessment of the patient.

Untreated compartment syndrome mediated ischemia of the muscles and nerves lead to eventual irreversible damage and death of the tissues within the compartment and as a long term result Volkmann's contracture.

## Case Presentation

We report the case of a 44-year-old healthy male tourist (no relevant medical history), who was bitten in the right hand by an adder during a getaway at the countryside of Denmark. Initial treatment was performed in a local county hospital in Denmark with analgesia and bandage. Due to persistent swelling and beginning lymphangitis, the patient was transferred next day to our University Hospital close to his residence. At the time of admission the main symptoms were significant swelling of the right hand, forearm and upper arm with lymphangitis up to the axilla.

**Figure 1:**
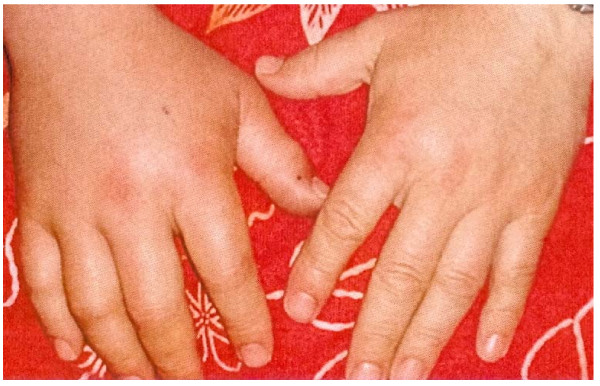
**44-year old male patient presented with adder bite in his right hand with beginning swelling, picture documentation immediately after bite by patient's relatives**.

The patient suffered from pain at a visual analogue scale around 7 (Scale 0-10). The patient reported beginning paresthesia of the median nerve. The palpation of the upper extremity revealed a hard swelling with a beginning compartment syndrome (Figure 1).

Laboratory tests showed a leucocytosis and elevated CRP with a body temperature of 38.1°C. The Antivenom (type European Viper Venon Antivenom) was immediately ordered and administered intravenous under ICU-conditions according to the guideline protocol, which recommend the early treatment within 48 hours. No complications occurred.

**Figure 2:**
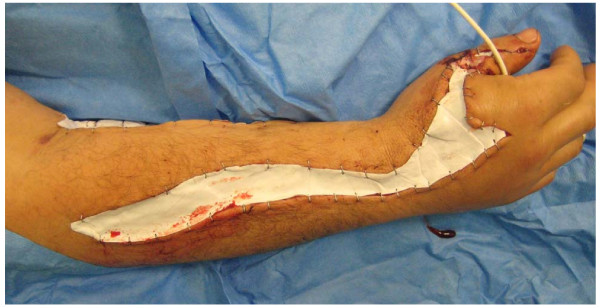
**intraoperative view of right forearm after compartment decompression (Epigard^® ^coverage over necrotic muscle tissue)**.

Only a few hours after admission of the patient in our hospital (before arrival of Antivenom) the surgical intervention with local incision of the loge of Guyon, the carpal canal, forearm and upper arm was performed (Figure 2). Intraoperatively, necrotic muscle tissue and hemorrhagic spots occurred. The wound area was temporally covered with Epigard^® ^(dermal substitute).

Post-op day 1 showed already a significant reduction of clinical signs of compartment syndrome.

After 4 days, the defect coverage was performed with secondary wound closure.

Long term follow up (1 year) showed sufficient wound healing, also the nerval function recovered completely with a full range of motion of all digits. He had returned to all previous activities and considered his hand and arm to be normal.

**Figure 3:**
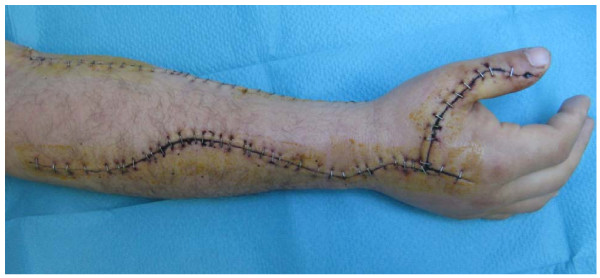
**postoperative view of right forearm of the patient (2 weeks post-op, healed wound)**.

Figure 3 shows a picture of the wound status 2 weeks post-op.

## Discussion

Snake venom poisoning is a medical emergency requiring immediate attention. Bites from poisonous European snakes can lead to local tissue damage and systemic symptoms [2]. The effects of envenoming are unpredictable and therefore victims should be referred to hospital for monitoring.

Treatment has two components: firstly correction of the systemic hemodynamic, respiratory and hematological disturbances and secondly administration of specific antivenom [14]. Envenomation of a limb can lead to cutaneous necrosis, compartment syndrome and even necrotising fascitis [9]. Early diagnosis and prompt treatment is needed to prevent these complications. Immediately after an adder bite, the bite site should be immobilized to delay the spread of the venom [15]. In case of a compartment syndrome, which can affects the whole upper extremity beside the carpal canal, the complete decompression of all compressed structures is ultimatively necessary in order to avoid further tissue damage and late sequalae [11]. Therefore we recommend that the classical signs and symptoms of compartment syndrome serve as indication for surgical decompression. Patients should be monitored for at least 24 hours with measurement of blood pressure, heart-, and respiratory rate as well as lab tests including white blood cell count, serum creatine kinase and bicarbonate. It is recommended that victims also should have an ECG twice daily if hypotension persists [8].

The Antivenom should be given, in case of systemic envenomation, intravenously at a dose of 20 ml (10 mg/ml) diluted with two to three volumes of normal saline and a rate not exceeding 2 ml of diluted antivenom per minute [10,16]. Reactions to antivenom are very rare; however, victims with allergic histories are at increased risk of developing severe antivenom reactions [8]. They should therefore only be given antivenom if there are definite signs of severe systemic envenoming, for example systolic hypotension < 80 mm Hg, coagulopathy, pulmonary edema, ECG abnormalities and peripheral leucocytosis > 15000/μl. A Sheep-fab-fragment antivenom, which is less allergenic than other antivenoms, should be employed in case of severe symptoms. The risk of reactions with currently available antivenoms for use in bites by European vipers is very low [10,17].

One study reported that in cases of severe envenoming the median duration of hospital admission was reduced from 10 days to 5 days in those receiving antivenom [18]. The overall mortality resulting from adder bites is very low and less common now than in the past, where for example in Sweden between 1920 and 1950 there were 24 reported deaths [7].

Any victim of snakebite presenting to a general practitioner or hospital for treatment in northern Europe can be assumed to have been bitten by the adder (V. berus), as this is the only naturally occurring venomous snake in these areas [1]. Although "exotic" venomous snakes are seldom kept as pets. Although envenoming is a likely result of receiving a bite from adder it is not inevitable, as "dry bites", in which no venom is injected, are known to occur in a number of tropical venomous snake species and may account for in excess of 50% of "accidental" bites in some species. Also, the quantity of venom injected can vary depending on the size of the snake, the efficiency of the bite, and the contents of the venom apparatus at the time of the bite [2]. In addition, intraspecific variation in venom components within the geographical range of a species or between individuals in the same location has been demonstrated in snakes from Australia and the tropics, resulting in a range of effects of envenoming in snake bite victims [19]. It is therefore likely that similar variations in the components of adder venom may also occur, resulting in unpredictable effects, thus underlining the need for adder bite victims to be monitored in hospital. Our case presentation deals purely with an adder bite and it is obligatory to state, that usually the dangerousness of tropical snakes is much higher.

In summary the recommended consensus protocol for the treatment of adder (V. berus) bite victims is that they should all be admitted to hospital and be observed and monitored for a minimum of two hours [8]. Any significant bite site should be excised locally under sterile surgical conditions. Asymptomatic cases may then be discharged. Overall the most cases of adder bites are mild, asymptomatic and rarely require intervention besides monitoring. Any victim showing any evidence of envenoming (grade 2-4) should continue to be observed and monitored for a minimum of 24 hours. This is especially important in case of children and the elderly, who are at particular risk from the effects of envenoming [8]. The antivenom should be given whenever there is any evidence of systemic envenoming or when local symptoms of envenoming are severe. During the administration of antivenom an injection of adrenaline should be immediately available for the treatment of anaphylactic antivenom reactions [8]. Reassurance of a victim is an important aspect of adder bite treatment. In conclusion effective treatment protocols can reduce both the length of time victims spend in hospital and the morbidity in the affected areas.

## Conclusions

Snakebite envenomation is an uncommon condition in the northern hemisphere, but requires high vigilance with regard to both the systemic effects of the venom and the locoregional impact on the soft tissues. Administration of antivenom and early surgical intervention is limb saving.

## Consent

Written informed consent was obtained from the patient for publication of this case report and any accompanying images. A copy of the written consent is available for review by the Editor-in-Chief of this journal.

## Competing interests

The authors declare that they have no competing interests.

## Authors' contributions

LHE have made the main substantial contributions to the idea, conception and design, acquisition of data, analysis and interpretation of data and have been mainly involved in drafting the manuscript and revising it critically for important intellectual content and have given final approval of the version to be published. TB participated in the whole case report and co-drafted the manuscript. TL participated in the whole clinical case. PM participated in the whole clinical case and coordination. All authors read and approved the final manuscript.
